# Evaluation of Cervical Mucosa in Transmission Bottleneck during Acute HIV-1 Infection Using a Cervical Tissue-Based Organ Culture

**DOI:** 10.1371/journal.pone.0032539

**Published:** 2012-03-07

**Authors:** Chengli Shen, Ming Ding, Deena Ratner, Ronald C. Montelaro, Yue Chen, Phalguni Gupta

**Affiliations:** 1 Department of Infectious Diseases and Microbiology, Graduate School of Public Health, University of Pittsburgh, Pittsburgh, Pennsylvania, United States of America; 2 Department of Infectious Diseases, Capital Medical University Beijing Youan Hospital, Beijing, China; 3 Department of Microbiology and Molecular Genetics and the Center Vaccine Research, University of Pittsburgh, Pittsburgh, Pennsylvania, United States of America; German Primate Center, Germany

## Abstract

**Background:**

Although there are different strains of HIV-1 in a chronically infected individual, only one or limited virus strains are successfully transmitted to a new individual. The reason for this “transmission bottleneck” is as yet unknown.

**Methodology/Principal Findings:**

A human cervical explant model was used to measure HIV-1 transmission efficiency of viral strains from chronic infections, and transmitter/founder variants. We also evaluated the genetic characteristics of HIV-1 variants in the inoculums compared to those transmitted across the cervical mucosa. Eight different HIV-1 isolates were used in this study, six chronic isolates and two transmitter/founder viruses. The transmission efficiency of the chronic and transmitter/founder virus isolates and the viral diversity of chronic isolates before and after viral transmission were assessed. The results indicate that transmitter/founder viruses did not display higher transmission efficiency than chronic HIV-1 isolates. Furthermore, no evidence for a difference in diversity was found between the inoculums and transmitted virus strains. Phylogenetic analysis indicated that the sequences of variants in the inoculums and those present in transmitted virus intermingled irrespective of co-receptor usage. In addition, the inoculum and transmitted variants had a similar pairwise distance distribution.

**Conclusion:**

There was no selection of a single or limited number of viral variants during HIV-1 transmission across the cervical mucosa in the organ culture model, indicating that the cervical mucosa alone may not produce the transmission bottleneck of HIV-1 infection observed *in vivo*.

## Introduction

Sexual transmission via genital mucosal epithelial surfaces accounts for more than 80% of HIV-1 infections [1]. A major gap in our understanding of the mechanism of HIV-1 sexual transmission is the delineation of molecular events occurring at early stages of infection. These events are critical in the establishment of local as well as systemic infection. Recently, by using single genome amplification (SGA) and ultra-deep sequencing methodologies, a number of investigators have reported that HIV-1 and SIV transmission occurs by a single or limited numbers of R5 tropic transmitted/founder virus variants with a very low diversity, even though the viral inoculums have a number of highly diverse variants [2], [3], [4], [5], [6], [7]. Currently the reasons for such variant selection during HIV-1 transmission, known as the transmission bottleneck, are undefined. There exists several possibilities to explain selection of a single or limited variants during transmission across the cervical/vaginal mucosa: a) the mucosa exerts a selection pressure on the transmitted/founder virus, b) there is competitive selection among transmitted variants as they traverse through mucosa to the regional lymph node and then to systemic compartments, or c) transmission of a single or limited variants from the mixture of quasispecies in the inoculums may occur in a stochastic fashion.

Evaluating the role of cervical mucosa at very early events of HIV-1 transmission is difficult to perform in humans. Although the SIV/macaque model has been used for this purpose, this animal model may not accurately reflect HIV-1 infection of humans at mucosal surfaces. To circumvent this problem, we have developed a cervical tissue-derived organ culture model to study HIV-1 transmission across the cervical mucosa isolated from women [8]. Unlike cell lines, this model provides the natural tissue architecture, including epithelial cells, submucosa, and immune cells such as T cells, macrophages (mΦ) and Langerhans cells (LC), and allows for the evaluation of transmission of infectious cell-free and cell-associated HIV-1 across the mucosa. Detailed studies have documented that the HIV-1 transmission observed in this organ culture are highly specific and not due to nonspecific leakiness in the tissue explant. These data include: i) lack of any discontinuity of the basal layer of the tissue during culture as assayed by transmission electron microscopy, ii) lack of transmission across the mucosa of blue dextran, a polysaccharide of 2×10^6^ Da molecular weight (smaller than HIV-1 virions) and fluorescent beads of 7.2 micron (similar size to HIV -1 particles), and iii) lack of mucosal transmission by UV and heat inactivated HIV-1 [8], [9] We have also examined the cervical tissue integrity during *in vitro* culture. Hematoxylin and eosin staining of the paraffin embedded tissue at various times after cultivation indicated that the stratified squamous epithelial layers and especially the basal layer of cells remained largely unchanged after culture [8]. The cellular functions of the tissues during the culture period were also assessed by quantitative immunohistochemical analysis for three immune cell markers (CD45RO for memory T lymphocytes, S100 for dendritic cells, and CD68 for macrophages), as well as two non-immune cellular markers (cytokeratin as a differentiation marker of the epithelial cells and Ki67 as a cell proliferation marker [10], [11], [12]). The levels of these five cellular markers remained unchanged during cultivation in both mock-exposed and HIV-1-exposed tissues, indicating the tissues are functionally active.

Using this cervical tissue-based organ culture we have shown that CD4+T cells are the first cells that become infected within 6 hr of exposure of cervical tissue to HIV-1. Thereafter, HIV-1 infected macrophages and dendritic cells were detected after 1 and 3 days of infection, respectively [9]. Using simultaneous *in situ* hybridization and immunophenotyping techniques, HIV-1 expressing CD4+ T lymphocytes, macrophages, and dendritic cells are detected at the intraepithelial layer within 3 days of infection, as observed in SIV/macaque system at early stages of infection [9]. Furthermore, the initial small pool of infected CD4+cells observed at 6 h post infection is amplified 10-fold within 24–96 h. Within this same time frame, more infected cells are detected in the submucosa and in the intraepithelial layer. Thus, this pattern of infection in the organ explant model is very similar to infection pattern reported in experimental infections of macaques by SIV [13], [14]. Finally, we and others have shown that HIV-1 transmission through cervical mucosa in the organ culture is inhibited by HIV-1 RT and entry inhibitors. These latter properties formed the basis for using this type of organ culture to evaluate various anti-HIV-1 microbicides by us and a number of investigators [6], [15], [16], [17], [18]. Therefore, the cervical tissue based organ culture can serve as a useful and informative model for HIV-1 transmission in humans.

In this report we have used this well established and validated organ culture model to delineate the role of cervical mucosa in selection of viral variants in HIV-1 transmission. Our data indicate that the cervical mucosa alone does not appear to have a significant role in the characteristic restrictive selection of HIV-1 variants that leads to the observed extremely low diversity of viral infection after mucosal exposure.

## Materials and Methods

### Ethics Statement

The cervical tissues for the organ culture were harvested from patients who undergo routine hysterectomy or anterior/posterior procedures at the Magee Women Hospital (MWH) of the University of Pittsburgh Medical Center. All tissues were obtained through the Tissue Procurement Facility of the hospital. This study was approved as an Exempt Study by the Institutional Review Board of the University of Pittsburgh. Individual informed consent was waived because this study used routinely procured tissue from patients through the Tissue Procurement Facility without any patient identification. No patient was specifically enrolled for this purpose. No specific racial or ethnic distribution was sought during the collection of tissue.

### Viruses

Eight different HIV-1 isolates were used in this study, including one HIV-1 subtype A (RW92008) from Africa, four HIV-1 subtype B (two R5 tropic and two X4 tropic), one HIV-1 subtype C R5 (IN93999) from India, and two transmitted/founder R5 viruses from an acute HIV-1 infection study (CH040 and CH058) [19]. Of the 4 subtype B isolates, two are laboratory strains (IIIB and BAL) and two are clinical isolates (015 and 074) that were obtained from the Multicenter AIDS Cohort Study (MACS). Africa subtype A (RW92008), Indian subtype C (IN93999) viral isolates, and two plasmids DNA containing the infectious molecular clones of transmitter/founder viruses (CH040 and CH058) were obtained from the NIH AIDS Research and Reference Reagent Program. Viruses from infectious clones were generated from transfection of 293T cells. All viruses were grown in PHA-stimulated CD8-depleted PBMC, and their infectivity titers were determined in CD8-depleted PBMC.

### Cervical Organ Culture

The organ culture was performed with cervical tissues from HIV negative premenopausal women, as previously described [8], [20]. The selected cervical tissues did not have any sign of precancerous lesions, and the donor women tested negative for sexually transmitted infections. Briefly, a 6 mm biopsied cervical tissue explant was placed into a transwell with the epithelial layer oriented upwards and sealed around its perimeter with 3% agarose. The tissue containing transwell was then placed into a 12-well plate containing 1 ml of RPMI-1640 media supplemented with 10% FBS. To measure the transmission across the cervical mucosa, cell-free HIV-1 in culture medium (BAL, IIIB, 015, 074, RW92008, IN93999, CH058C or CH040C with TCID_50_ values described in Table 1) was added to the top of the tissue in the upper chamber. In the case of mixed infections (e.g., HIV-1 BAL and transmitter/founder viruses or HIV-1 BAL and EIAV), the inoculums of transmitting pairs were diluted appropriately to achieve 1∶1 ratio of the two strains with respect to TCID_50_ or viral RNA copies. A transwell with the membrane only served as a positive control, while a transwell with agarose only served as a negative control. Cultures were incubated at 37°C for 3 days in a CO_2_ incubator. Transmission from each virus was tested in tissues from 1–2 donors with at least triplicate tissue biopsies from each tissue donor. On the third day, the tissue was removed, and the culture supernatants in the bottom chamber and the original viral inoculum were centrifuged at high speed (22,000 rpm) in a Biofuge Stratos centrifuge (Sorvall Instrument) to pellet virions. RNA was then extracted from the pelleted virus for the analyses of viral RNA concentration and diversity, before and after viral transmission. The levels of strain specific HIV-1 RNA species in single or mixed infections were quantitated using TaqMan® real-time PCR using primers specific for the respective viral strain. To ensure that transmitted viruses were not due to leaks in the system, the intactness of the tissues were evaluated at the end of each experiment by examining the transmission of blue dextran through the tissue and agarose control wells. These assays confirmed that the amount of blue dextran transmitted through the tissue well was equal or lower than that in the agarose control well. The transmission efficiency through cervical explants was calculated by dividing the number of viral RNA copies that were detected in the bottom well supernatant by the number of viral RNA copies detected in the supernatant of the membrane-only well and multiplying by 100%.

**Table 1:** Characteristics of Viral Inoculums Used in the HIV-1 Transmission and Diversity Studies.

Viral Isolates	Origin of Isolate	Co-Receptor Usage	Clade	Viral Inoculum (TCID_50_)
IIIB	Laboratory	X4	B	1×10^5^
Bal	Laboratory	R5	B	1×10^6^
015	Clinical	R5	B	1.2×10^5^
074	Clinical	X4	B	6.2×10^3^
CH058	Transmitter/Founder	R5	B	3.1×10^4^
CH040	Transmitter/Founder	R5	B	6.2×10^3^
RW92008	African	X4	A	8.1×10^3^
IN93999	Indian	R5	C	2.5×10^2^

### Sequence analysis

For sequence analysis, total RNA was extracted from the viral pellets and subjected to reverse transcription. The primer for reverse transcription was ED12 5′-AGTGCTTCCTGCTGCTCCCAAGAACCCAAG (7782–7811*), and the primers for nested PCR were: first round forward primer: ED31 5′-CCTCAGCCATTACACAGGCCTGTCCAAAG (6811–6845*), reverse primer: BH2 5′-CCTTGGTGGGTGCTACTCCTAATGGTTCA (7725–7697*), second round forward primer: DR7 5′-TCAACTCAACTGCTGTTAAATGGCAGTCTAGC (6990–7021*), reverse primer: DR8 5-CACTTCTCCAATTGTCCCTCATATCTCCTCC (7668–7638*). (*the positions are based on HIV-1 HXB2 strain). Single genome amplification was performed as described previously [3], [5]. Amplicons were directly sequenced in an ABI Prism 3700 DNA Sequencer. Vector NTI software was used to inspect the chromatograms. The sequences without mixed bases were taken as an evidence of single genome amplification from a single viral RNA/cDNA template. DNA alignments were constructed using CLUSTAL X alignment program and hand adjusted when necessary. The sequences were visually inspected by using neighbor-joining phylogenies implemented in MEGA5.0 [21] to test the potential bottleneck during the variants across the mucosa.

Two quantitative methods, Slatkin-Maddison and diversity analysis were further employed to evaluate the effect of the cervical mucosa on viral transmission. A cladistic method of Slatkin-Maddison analysis [22], [23] implemented in the HyPhy [24] program was used to assess the possible enrichment of the transmitted HIV variants. A phylogenetic tree was constructed for each inoculum and transmitted viral pairs. The number of migration events needed to postulate the observed spatial distribution of HIV-1 sequences in the phylogenetic trees was estimated. The null model of this analysis is that all the sequences from the transmitted variants would be as likely to be related to the inoculum as to themselves. The frequency of distribution under this null model was obtained by constructing 10,000 random trees made by random joining/splitting of the ‘true’ phylogenetic tree. The number of migration events on the true tree was then compared to the null distribution, and the probability that the true tree came from a population lacking similarity was determined. If there was variant selection during transmission across the mucosa and if the similar number of the inoculum and transmitted viral sequences were randomly selected, the transmitted viral sequences should trend to cluster together. Also, the number of migration events should be smaller compared with the random joining/splitting phylogenetic tree. The tests were performed with all viral sequences and after the removal of identical sequences, as these sequences can increase both the frequency of compartmentalization calls and the statistical support for those calls. The sequence enrichment was considered statistically significant if fewer steps were seen in the true tree than in 95% of the random trees. In addition, the neighbor-joining method was utilized for the diversity analysis as measured in MEGA5.0. Statistical comparisons of diversity between the inoculum and the transmitted variants were performed using the two-sample tests (Tsubjmean test) for comparing intra-individual sequence diversity between populations http://www.scharp.org/users/adecamp/diverstest/runtests.php [25]).

### Sequence Data

The HIV-1 *env* nucleotide sequence data were deposited in the Genbank nucleotide sequence.

Databases using Sequin V5.35 (http://www.ncbi.nlm.nih.gov/Sequin/), under the following.

Accession numbers: JN415236–JN415469.

## Results

### Comparison of transmission efficiency between the founder/transmitter and chronic HIV-1 isolates

In our previous studies we have provided substantial evidence that HIV-1 transmission observed in the organ culture is highly specific and cannot be attributed to leakiness in the system. To further substantiate the specificity of HIV-1 transmission in this organ culture model, we compared the transmission of HIV-1 BAL with equine infectious anemia virus (EIAV), an equine lentivirus similar in size to HIV, but unable to infect human cells. Thus, the EIAV serves as a highly relevant control for nonspecific transmission across human cervical mucosa tissues. As shown in Table 2, exposure of the cervical tissue explants to EIAV produced a viral transmission level that was less than 0.0001%, a value that was similar to the EIAV transmission observed in the agarose control, indicating a lack of specific transmission of the EIAV through the cervical tissue. In marked contrast, exposure of the cervical tissue explants to an HIV-1 BAL yielded a transmission efficiency of 0.32%, or about 1000-fold higher than observed with EIAV, despite the use of similar inoculum doses of 5×10^7^ RNA copies of each virus. Furthermore, when a mixed inoculum of EIAV and HIV-1 at a ratio of 1∶1 (by RNA content) were used to infect the cervical explant, the transmission efficiency of EIAV was observed to be less than 0.0002% compared to the HIV-1 BAL transmission efficiency of 0.25%. Taken together, these results conclusively demonstrate that HIV-1 transmission in the cervical tissue based organ culture is highly specific therefore, can be reliably used to measure transmission of HIV-1 across cervical mucosa [8].

**Table 2:** Transmission of EIAV and HIV across cervical mucosa in an organ culture.

Vial Inoculum	Transmission efficiency(%)*
HIV BAL	0.32
EIAV	<0.0001
Agarose control for HIV	<0.0001
Agarose control for EIAV	<0.0001
HIV+EIAV (1∶1)	
HIV	0.25
EIAV	<0.0002

*Denotes transmission efficiency of HIV or EIAV RNA from a mixture of EIAV and HIV(1∶1) inoculum. Transmission efficiency was calculated as described in the materials and methods section.

**Figure 1:**
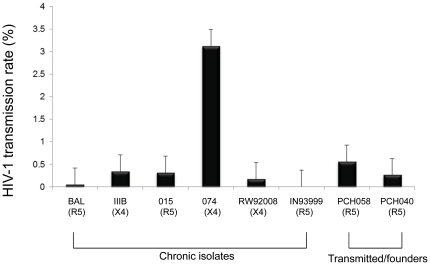
The transmission efficiencies of various HIV-1 inoculums across the cervical mucosa. The transmission efficiencies were calculated as the percentage of the viral load in the inoculums that were transmitted across cervical mucosa. X axis denotes the isolates (co-receptor usage indicated in the parenthesis) used in this study while Y axis denotes the percentage of the transmission. Data shown here is the average of 3 biopsies with standard error. Isolates are grouped as chronic isolates and transmitter/founders.

Having validated the specificity of HIV-1 transmission in the organ culture system, we next sought to evaluate the role of cervical mucosa during HIV-1 transmission by comparing the transmission efficiencies of reference clinical isolates from different HIV clades (A, B, and C), of different tropisms (R5 or X4), and of transmitted/founder viruses characterized in an acute infection study [19]. The dose of the inoculum for each virus isolate was shown in Table 1. Using the cervical organ culture model, the transmission efficiency of each virus strain was determined as described in Materials and Methods. As shown in Figure 1, the transmission efficiency of the six reference clinical isolates ranged from 0.005% to 3.12% (median 0.332%). Although there were variations in transmission among different isolates, there were no apparent significant differences in transmission efficiency levels among different virus isolates that are R5 and X4. Furthermore, the results indicate that the viral inoculum size does not have a significant influence on the observed transmission efficiency (compare Table 1 and Figure 1). For example, the highest transmission efficiency (3.12%) was observed with the 074 clinical isolate at a dose of 6.2×10^3^ TCID_50_, while one of the lowest transmission efficiencies (0.332%) was observed with the BAL isolate at a higher dose of 1×10^6^ TCID_50_.

Since the transmission properties of related transmitted/founder viruses are of great interest to understand HIV-1 transmission, we examined whether the transmitted/founder viruses reported in an acute HIV-1 infection study [19] have any selective advantage of viral transmission across the cervical mucosa. For this purpose, we first examined the viral transmission efficiency individually of the two transmitted/founder viruses CH040 and CH058. The transmission efficiency of the two transmitted/founder variants (CH040 and CH058) were 0.56% and 0.26%, respectively (Figure 1). These transmission efficiency values are similar to the transmission efficiency levels observed above for chronic and lab viral isolates. Therefore, the transmitted/founder viruses do not appear to have higher transmission efficiency than those non- transmitted/founder HIV-1 variants.

Next, we examined whether CH040 and CH058, being transmitted/founder viruses, have a transmission advantage over a non- transmitted/founder HIV-1, such as HIV-1 BAL, in a competitive transmission assay, as described previously [20]. For this purpose, equal TCID_50_ amounts of CH040 or CH058 were mixed with HIV-1 BAL and used as inoculum in the organ culture system. Transmitted HIV-1 contained in culture supernatant was analyzed for the relative levels of CH040 or CH058 and BAL viruses by a real-time PCR using the primers/probes set that can discriminate between transmitted/founder virus and BAL. As shown in Table 3, these results from the BAL/CH040 pairwise competition indicated that transmission efficiencies were 1.72% for HIV-1 BAL and 1.68% for the transmitted/founder strain CH040. Similarly, in the BAL/CH058 pairwise competition assay, the transmission efficiency for HIV-1 BAL was 0.44%, while the transmission efficiency for the transmitted/founder strain CH058 was 0.63%. Thus, the transmitted/founder viruses do not appear to have any competitive advantage over other viral isolates in their transmission efficiency through cervical mucosa.

**Table 3:** The competition analysis of the transmission efficiency for the transmitted/founder viruses and chronic HIV-1 isolates.

Viral pairs	Variant(co-receptor)	Inoculum viruses (TCID50)	Transmission efficiency*
**BAL and CH040**	BAL (R5)	3.0×10^5^	1.72%
	CH040(R5)	1.0×10^6^	1.68%
**BAL and CH058**	BAL (R5)	1.0×10^4^	0.44%
	CH058(R5)	0.6×10^4^	0.63%

HIV-1 CH040 and CH058 are transmitted/founder viruses and HIV-1 BAL is a chronic isolate.

*The transmission efficiency was calculated by dividing the viral RNA copies in the inoculum with the viral RNA copies of the transmitted variants from the bottom wells. Real-time RT PCR with specific primers/probes set which could discriminate CH040, CH058 and BAL viruses was employed.

### Genetic analysis of HIV-1 variants in viral inoculum and those present in transmitted virus

**Figure 2:**
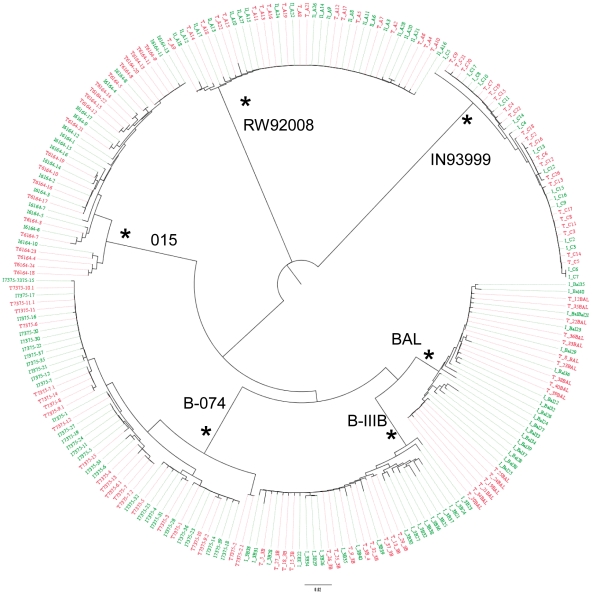
Phylogenetic analysis of all viral inoculum and transmitted pairs. C2-V5 of *env* nucleotide sequences of the inoculum and transmitted HIV-1 species were aligned for all linked inoculum variants (green lines) and transmitted variants (red lines) and a neighbor-joining tree representing different transmission pairs. Branch lengths are drawn to scale. Viral strain names are shown on the branches, and asterisks are indicated on branches with bootstrap values greater than 99%.

To evaluate transmission characteristics of transmitted HIV-1 variants, we analyzed HIV-1 sequences of transmitted viruses across the cervical mucosa using SGA to minimize the potential for template switching during the PCR reaction and re-sampling because of unequal template amplification and cloning *in vitro* [5]. Each amplicon was directly sequenced in the variable C2–V5 region of HIV-1 envelope. A total of 230 *env* C2-V5 gene sequences were generated by SGA from the starting inoculums and from the viruses transmitted across the cervical mucosa. A median of 18 sequences per isolate (range 15–20) was used for subsequent analysis. Neighbor-joining phylogenetic trees were constructed using the sequences from all six inoculum/transmission pairs. For all of the variants, the inoculum sequences were heterogeneous, consistent with their derivation from chronically infected individuals (Figure 2). Importantly, the transmitted variant sequences (red) were quite similar to that of the inoculum variant sequences (green), which were heterogeneous and intermingled in the inoculum sequences (Figure 2). These data clearly indicate that multiple inoculum quasispecies were transmitted through the cervical mucosa in the organ culture.

**Figure 3:**
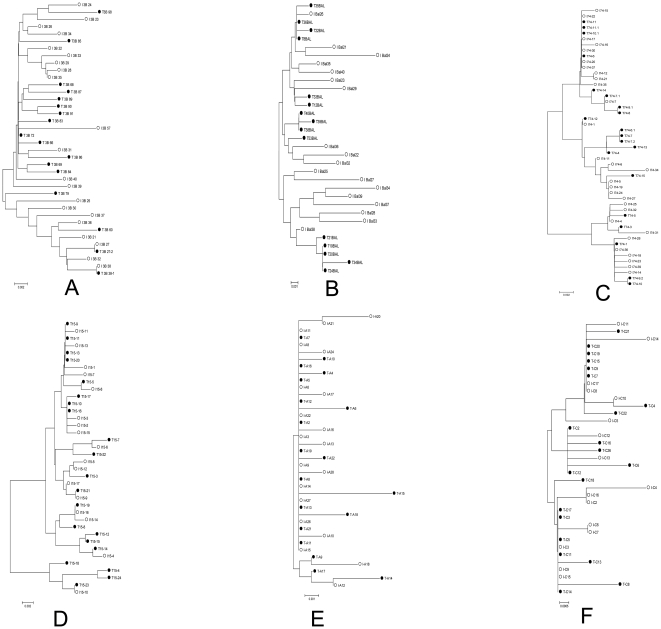
Neighbor-joining trees of HIV-1 env C2-V5 sequences individually from six inoculum and transmission pairs. Aligned nucleotide sequences for six inoculums and transmitted variants were used to generate neighbor-joining trees for individual viral pairs. (A) HIV-1 subtype B, IIIB; (B) HIV-1 subtype B, BAL; (C) HIV-1 subtype B, clinical isolate 074; (D) HIV-1 subtype B, clinical isolate 015; (E) HIV-1 subtype A, RW92008; (F) HIV-1 subtype C, IN93999. White circles: the sequences of the inoculum viruses; black circles: the sequences of the transmitted viruses.

We then examined the phylogenetic relationships of *env* sequences between viral inoculums and their corresponding transmitted viruses individually in a neighbor-joining tree (Figure 3). When HIV-1 IIIB was used as the inoculum, the transmitted viral sequences (black dots) were intermingled with those of the inoculum quasipecies (black circles), and almost all the lineages in the inoculum could be found in the transmitted variants (Figure 3A). The phylogenetic analysis was then extended to the paired sequences of HIV-1 variants in inoculums and those present in transmitted viruses for HIV-1 BAL (Figure 3B), clinical isolate 015 (R5) (Figure 3C), clinical isolate 074 (X4) (Figure 3D), the subtype A RW92008 (Figure 3E) and the subtype C IN93999 (Figure 3F). The results were quite similar to that of HIV-1 IIIB in that the phylogenetic analyses of all of these pairs failed to detect distinct genetic differences between the inoculums and the transmitted viral variants. Taken together, these phyologentice studies indicate a lack of selection in the transmission of diverse laboratory and clinical HIV strains across the cervical mucosa.

### Diversity analysis of the inoculums and transmitted HIV-1 variants

**Figure 4:**
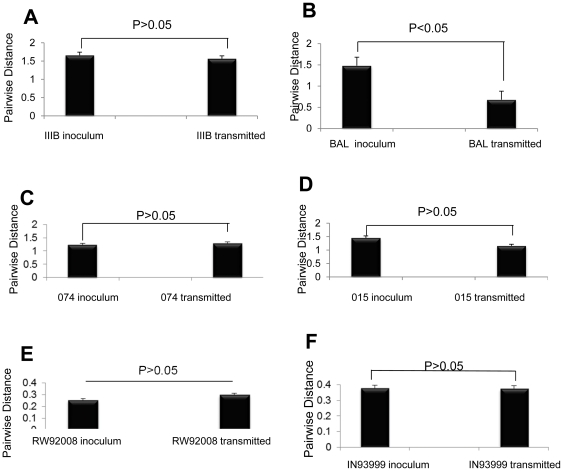
Diversity analysis of the six HIV-1 isolates before and after their mucosal transmission. Diversity analysis of envelope sequences (C2–V5) from different isolates was performed using MEGA5.0 software. Nucleic acid intra-population diversity was calculated based on nucleotide sequences. The differences between the diversity were plotted as intra-population distances as a function of inoculums and transmitted viruses. Statistical significance of the diversity differences between the inoculums and transmitted viruses was performed using two-sample tests [25]. (A) HIV-1 subtype B, IIIB; (B) HIV-1 subtype B, BAL; (C) HIV-1 subtype B, clinical isolate 074; (D) HIV-1 subtype B, clinical isolate 015, (E) HIV-1 subtype A, RW92008; (F) HIV-1 subtype C isolate, IN93999.

To further compare the genetic characteristics of viral variants in inoculums and transmitted virus populations, the viral diversity analysis was also performed according to the paired sequence distances. Mean intra-viral diversity ranged from 0.2% to 1.45% for the inoculums and from 0.2% to1.6% for the transmitted variants, respectively (Figure 4). Viral diversity was not statistically significant (p>0.05) between the inoculums and transmitted viruses (Figure 4A–F) in 5 of 6 isolates, regardless of their subtypes and co-receptor usage. However, in the case of HIV-1 BAL, the diversity of transmitted virus was significantly decreased compared to that of the inoculums (Figure 4B), but it was still much higher than one would have expected from the transmission of a single viral variant, as observed in human and monkey infections [3], [4]. Although the diversity of the subtype A and C inoculums (Figure 4E, F) was lower compared to that of subtype B, there was still no significant difference in diversity between inoculums virus and transmitted virus populations. In addition, a cladistic method of Slatkin-Maddison analysis [22], [23] was also used to assess the possible enrichment of the transmitted HIV variants. The tests were performed with all viral sequences, and after removal of identical sequences. The results of Slatkin-Maddison analyses are shown in Table S1. These data indicate no significant enrichment in the transmitted viral strains in 4 of 6 isolates. However, for HIV-1 BAL there was a significant trend of enrichment in transmitted variants, regardless whether the identical sequences were removed or not for the analysis. For HIV-1 074 strain, there was a significant trend of enrichment in the transmitted virus when the test was performed with all viral sequences, but, the enrichment was not significant when the identical sequences were removed (Table S1). These data demonstrate that the diversity and Slatkin-Maddison analyses yielded comparable conclusions. Although there was a 10–100 fold difference in infectivity of viral inoculums used for the different viral isolates (Table 1), the current analyses did not reveal extreme low diversity in any of the virus populations transmitted in our organ culture system, a result that is in distinct contrast to the low diversity detected in viral populations transmitted in humans or monkeys [2], [3], [4], [5], [6].

To further examine the role of inoculum size on the selection of transmitted virus and hence diversity in the transmitted virus population, we inoculated cervical tissue in our organ culture with a serial half log decreasing dose of HIV-1 BAL and measured viral diversity in the transmitted virus population. Each inoculum was tested in 3 biopsies, and the whole experiment was conducted with biopsies from a single donor to rule out possible effects from patient variation. The results shown in Table 4 indicate that the transmission rate is quite similar with all inoculums and that there is no significant difference in viral diversity between inoculums and the transmitted variants generated from the different dilutions of inoculum.

**Table 4:** Measurement of the transmitted HIV-1 IIIB diversity from serially diluted viral inoculums.

Viral inoculum(TCID50)	% of transmission	Viral diversity(mean±SD)%*
3×10^5^	1.12	1.14±0.13
1×10^5^	0.04	1.23±0.15
3×10^4^	0.97	1.13±0.12

The viral diversity of the viral inoculum is 1.20±0.09%.

*No significant difference in viral diversity between the inoculum and all the three different transmitted variants.

**Figure 5:**
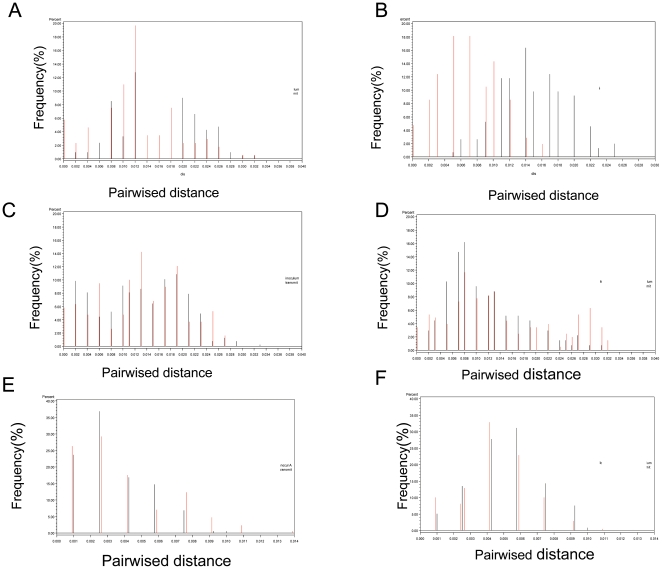
Comparison of distance distribution between the inoculums and the transmitted viral sequences. The distance distribution for the inoculums and transmitted pairs was shown in pairwise distance histogram. For each pair, the distances between every possibility of pair sequences of inoculums and transmitted variants were calculated respectively. (A) HIV-1 subtype B, IIIB; (B) HIV-1 subtype B, BAL; (C) HIV-1 subtype B, clinical isolate 074; (D) HIV-1 subtype B, clinical isolate 015; (E) HIV-1 subtype A, RW92008; (F) HIV-1 subtype C, IN93999. Black needle bars represent the inoculums pairwise viral sequence distance frequency. Red needle bars represented the transmitted pairwise viral sequence distance frequency.

The analysis of pairwise distance distribution of the transmitted variants can reflect the transmitted viral genetic characteristics and display the effect of the minor diverse variants when comparing the paired viral diversity. In general, the inoculum and transmitted pairs displayed a similar pairwise distribution (Figure 5A–F) indicating a lack of selection in the transmission process. In the case of the BAL infection, however, the transmitted viral diversity shifted slightly left (Figure 5B), indicating that there was a trend towards selection during BAL transmission. However, according to the distribution, there were still multiple quasispecies in the transmitted of BAL variants.

## Discussion

Previous studies [4], [6] with acutely infected monkeys demonstrated that from an inoculum containing numerous SIV viral variants, only one quasispiecies or a limited number of variants crossed the cervical mucosa and established infection in the recipient host. Similar transmission of a single R5 tropic HIV-1 variant has also been reported during sexually transmitted infection in human [3]. The mechanism for transmission of single or a limited number of HIV-1 variant viruses is not clear. We reported earlier that cervical mucosal organ culture could serve as model to study HIV-1 transmission across cervical mucosa [8], [9] .There are potential limitations in using organ culture to study the role of cervical mucosa in the transmission bottleneck of HIV-1: 1) an adaptive immune response acting on behalf of both partners in the transmission event is absent in organ culture, 2) mucosal secretions often present *in vivo* on cervical surface are mostly absent in organ culture. These secretions can be important because of their capacity to trap or inactivate infecting virions. However, transmission profiles using the organ culture are remarkably similar to those reported for SIV/macaque system with regard to types of cells that become infected and their localization in cervical tissue, and the expansion of the initial pool of infected cells during early stages of infection [13], [14]. Thus, we have used the organ culture to study the role of cervical mucosa in HIV-1 transmission bottleneck infection, because both the inoculum and transmitted variants can be accurately analyzed. Furthermore, by using the organ culture, we can focus on the specific role of the mucosal tissue in HIV-1 transmission bottleneck without confounding factors such as mucus, immune responses, etc. Sequence analysis of the transmitted virus isolated from the acute phase of infection in monkey or human indicated that the transmitted variants formed unique monophyletic or limited clusters in the phylogenetic tree [3], [4], [6]. In these studies it has been speculated that the mucosa might play an important role in viral variant selection. However, in the study reported here, phylogenetic trees (Figures 2 and 3) clearly demonstrated that the inoculum and the transmitter variants were intermingled, suggesting that most of the inoculum variants were transmitted across the cervical mucosa. The diversity and pairwise distance distribution analysis (Figures 4 and 5) indicated no statistical differences between the inoculum and transmitted variant populations. The current results also indicate that the lack of difference in diversity between the inoculum and transmitted variants is not due to inoculum size (Table 4). Consequently, the number of founders in our study was much higher than the limited number of founder virus reported *in vivo* [3], [4], [19]. These results support the idea that mucosa alone may not play a major role in genetic bottle neck observed during acute HIV-1 transmission.

This notion was further verified by comparing the transmission efficiency of HIV-1 isolates both from chronically infected subjects and from the transmitted/founder HIV-1 obtained during the acute stage of infection. Our data on the transmission efficiency among these isolates and direct competition analyses indicate that the transmission efficiency both of the chronic variants and the transmitted/founder variants were quite low, and that the founder/transmitter variants did not have a selective advantage of transmission over chronic HIV-1 isolates. Since each isolate was tested in different tissues in triplicate, the source of tissue did not seem to influence the efficiency of transmission. The low transmission efficiency levels observed in our organ culture system are consistent with the low transmission (0.01 to 0.001%) observed in SIV/macaque model, as reported by Miller et al. [26].

The current study using the organ culture suggests that cervical mucosa alone may not play a role in the transmission bottleneck observed *in vivo*. Furthermore, since most of the HIV-1 sequences in semen, the major vehicle in HIV-1 transmission are R5 tropic [27], [28], [29], [30], [31] and transmitting donors are mostly asymptomatic, it is expected that transmitting HIV-1 would be predominantly R5 tropic. Therefore, there may not be a need for any special mechanism for the transmission of R5 tropic viruses, as suggested by some investigators [32], [33], [34]. In addition, previous studies did not find any stretch of signature sequences in transmitted viruses [2], [3], [4], [5], [6]. Therefore, HIV-1 transmission of a single variant of R5 with no signature sequence can be best explained by the idea that HIV/SIV transmission is mostly a stochastic phenomenon. This was supported by previous reports which concluded that all of the sequences in the inoculum are probable to be transmitted to the recipient [4], [6]. However, a recent study on the analysis of HIV-1 variants of transmitter-recipient pairs indicates selection for a transmitting virus variant with a set of essential biological properties, such as fewer glycosylation sites, more compact *env*s, and a histidine residue at position 12 of the leader sequence of the *env* protein [35]. Therefore, other possibilities should also be considered: (i) it is possible that the mucosal immune system acts as a net, and the immune environment of the explant is different from *in vivo* infection; and (ii). The selection of viral variants occurs at the regional draining lymph nodes after crossing the cervical mucosal. Further studies related to the genetic bottleneck during HIV-1 and SIV transmission are warranted to support such a contention.

All of the founder virus-related studies with SIV reported in the literature [4] and the current HIV transmission studies have been performed with cell-free virus. However, transmission could occur via both cell-free and cell associated HIV-1/SIV, although transmission via cell-associated SIV was found to be much less efficient than cell-free virus [36], [37]. It is difficult to predict accurately the outcome of founder virus from cell-associated virus. Sexual transmission of HIV-1 mostly occurs via semen which contains both cell-free and cell-associated HIV-1. Since a limited number of founder virus strains were observed in sexually transmitted HIV-1 presumably via semen, a limited number of virus transmissions from cell-associated HIV-1 probably occur in a similar manner to cell-free HIV-1. Further study with cell-associated SIV in a macaque model would be useful to further define the pattern of cell-associated virus transmission compared to cell free virus transmission.

## Supporting Information

Table S1 Phylogenetic compartmentalization analysis using Slatkin-Maddison method.
(DOCX)
