# Supplementary material for: Evaluation of Cervical Mucosa in Transmission Bottleneck during Acute HIV-1 Infection Using a Cervical Tissue-Based Organ Culture
Source: PLoS One. 2012 Mar 7;7(3):e32539. doi: 10.1371/journal.pone.0032539 (PMC3296723; doi:10.1371/journal.pone.0032539)
Supplement: Table S1 — Phylogenetic compartmentalization analysis using Slatkin-Maddison method. (DOCX) [file pone.0032539.s001.docx]

| Viral Isolated | Migration Events^1^ | P value^1^ | Migration Events^2^ | P value^2^ |
| --- | --- | --- | --- | --- |
| IIIB | 10 | 0.0866 | 9 | 0.062 |
| Bal | 6 | 0.0011* | 6 | 0.0057* |
| 015 | 14 | 0.8757 | 9 | 0.2317 |
| 074 | 11 | 0.0089* | 7 | 0.0519 |
| RW92008 | 14 | 0.7614 | 5 | 0.1142 |
| IN93999 | 13 | 0.7903 | 7 | 0.5558 |

Table S1 Phylogenetic compartmentalization analysis using Slatkin-Maddison method

^1^The tests were performed with all viral sequences; ^2^The tests were performed after removing the identical sequences.

*P<0.05, significant compartmentalization between the inoculum and the transmitted viral sequences. There are enrichment in the transmitted viral sequences.
